# Reduction of Mental Health–Related Emergency Department Admissions for Youth and Young Adults Following a Remote Intensive Outpatient Program: Quality Improvement Analysis

**DOI:** 10.2196/47895

**Published:** 2023-11-09

**Authors:** Kate Gliske, Jaime Ballard, Katie R Berry, Michael Killian, Elizabeth Kroll, Caroline Fenkel

**Affiliations:** 1 Charlie Health Inc Bozeman, MT United States; 2 Center For Applied Research and Educational Improvement University of Minnesota Saint Paul, MN United States; 3 College of Social Work Florida State University Tallahassee, FL United States

**Keywords:** mental health, emergency room, emergency department, ED, readmission, intensive outpatient treatment, IOP, pediatric, youth, young adult, emergency, evaluation, readmission, sexual identity, race, ethnicity, care, outpatient, treatment

## Abstract

**Background:**

Pediatric mental health emergency department (ED) visits are increasing at 6% to 10% per year, at substantial cost, while 13% of youth with psychiatric hospitalizations are readmitted in the following weeks. Hospitals do not have the resources to meet escalating youth’s mental health needs. Intensive outpatient (IOP) programs, which provide multiple hours of care each week, have the power to reduce the number of patients in need of hospitalized care and provide a step-down option for patients discharging from ED’s in order to prevent readmissions.

**Objective:**

The purpose of this program evaluation was to assess (1) whether youth and young adult ED admission rates decreased following participation in a remote IOP program and (2) whether there were differences in readmission rates between youth and young adults by gender identity, sexual orientation, race, or ethnicity.

**Methods:**

Data were collected from intake and 3-month postdischarge surveys for 735 clients who attended at least 6 sessions of a remote IOP program for youth and young adults. Patients reported if they had been admitted to an ED within the previous 30 days and the admission reason. Over half (407/707, 57.6%) of clients were adolescents and the rest were young adults (300/707, 42.4%; mean age 18.25, SD 4.94 years). The sample was diverse in gender identity (329/687, 47.9% female; 196/687, 28.5% male; and 65/669, 9.7% nonbinary) and sexual orientation (248/635, 39.1% heterosexual; 137/635, 21.6% bisexual; 80/635, 10.9% pansexual; and 170/635, 26.8% other sexual orientation) and represented several racial (9/481, 1.9% Asian; 48/481, 10% Black; 9/481, 1.9% Indigenous; 380/481, 79% White; and 35/481, 7.2% other) and ethnic identities (112/455, 24.6% Hispanic and 28/455, 6.2% other ethnic identity).

**Results:**

Mental health–related ED admissions significantly decreased between intake and 3 months after discharge, such that 94% (65/69) of clients with a recent history of mental health–related ED admissions at IOP intake reported no mental health–related ED admissions at 3 months after discharge from treatment (*χ*^2^_1_=38.8, *P<*.001). There were no differences in ED admissions at intake or in improvement at 3 months after discharge by age, gender, sexuality, race, or ethnicity.

**Conclusions:**

This study documents a decrease in ED admissions between intake and 3 months after discharge among both youth and young adults who engage in IOP care following ED visits. The similar outcomes across demographic groups indicate that youth and young adults experience similar decreases after the current tracks of programming. Future research could conduct a full return-on-investment analysis for intensive mental health services for youth and young adults.

## Introduction

### Background

The youth and young adult mental health crisis has reached a state of emergency, according to the US General Surgeon [1]. The rate of emergency department (ED) use among youth experiencing mental health issues has increased 6% to 10% per year in the United States over the past decade, far exceeding historical national trends and outpacing the rise in nonmental health related pediatric ED visits [2]. Costs of care have also escalated [3], and pediatric mental health hospitalizations cost US $2 billion per year on average from 2006 to 2011 [4]. Further, there is concern that this increase in ED use for pediatric mental health reasons has only increased preexisting health disparities that affect historically disadvantaged communities such as marginalized racial and ethnic groups; those relying on public health insurance; and the lesbian, gay, bisexual, transgender, queer, intersex, or asexual community [2,5].

### Rise in Mental Health Related ED Utilization Rates

Pediatric mental health ED visits have been increasing for many years. Lo and colleagues [6] found that between 2007 and 2016, pediatric mental health ED visits rose 60%, while general pediatric ED visits remained unchanged. Similar increases were found in 2 additional studies of national data sets between 2006 and 2015, finding increases ranging from 48% to 54% [7,8]. The proportion of ED visits for mental health conditions further increased following COVID-19 [9].

Faced with escalating needs for mental health care, hospitals have insufficient resources to efficiently meet the needs of youth who go to the ED for mental health crises. The length of ED visits has increased, while the length of stay for other pediatric ED visits remained stable or increased only slightly [2,10]. In 2020, a large pediatric hospital noted that more than half of pediatric patients who visited the ED with a mental health concern needed to wait more than 2 days in the ED before being discharged, admitted, or transitioned to another facility [11], which is double the wait time of the previous year. During ED visits for mental health concerns, many youth never receive a mental health evaluation, likely due to limited staffing and inadequate evaluation protocols [12]. Approximately 30% of youth who visit the ED for a mental health concern are admitted to a hospital for psychiatric care [12,13], though researchers suggest many of these cases might have been managed with outpatient treatment if such resources were available [13]. The surge in ED visits and the challenges in meeting youth’s needs in the ED highlight both increasing youth’s mental health needs as well as the current lack of sufficient community mental health resources to address these needs [14].

### Hospital Readmissions

#### Overview

Many of these pediatric mental health ED visits are repeat visits by the same youth, whose mental health needs have not been adequately addressed. In a meta-analysis of youth’s psychiatric hospitalizations, 13.2% of youth were readmitted during the follow-up period [15]. The national average state hospital psychiatric readmission rates for young adults are in a similar range, with a 30-day civic readmission rate of 7.8% and a 180-day readmission rate of 15.6% [16]. Most readmissions occurred within 90 days of initial admission [17].

The costs of psychiatric hospital readmissions are substantial, as hospital costs for readmissions for mental health disorders are 22% higher than first admissions [18]. The Health Care Cost Institute [19] calculated the average price of a mental health admission as US $9879 (US $11,305 in 2023 dollars after adjusting for medical inflation using the US Bureau of Labor Statistics annual medical inflation rates [20]). Charges and costs of care vary widely by payer and by disorder. A thorough review of costs, charges, and payments for inpatient psychiatric treatment in community hospitals found that charges ranged from US $8393 for a stay for depression treatment for an uninsured client to US $20,937 for a schizophrenia treatment stay for a client on Medicaid in 2006 [21]. The exacerbation of illness [22], inadequate treatment during the hospital admission, and limited access to outpatient treatment are frequently cited as factors leading to readmission [23].

#### Factors Impacting Youth’s Readmissions

Clinical severity is predictive of readmission, more so than sociodemographic characteristics. Previous suicidal ideation and psychotic disorders are associated with increased risk of readmission, as well as prior hospitalization and discharge to residential services [15]. Similarly, individuals with comorbid conditions have a higher risk of readmission [24-26]. In a meta-analysis of pediatric psychiatric readmission, demographic variables, such as gender and age, did not have a direct effect on readmission [15,27], although 1 study concluded that demographics may interact with other variables [15]. Additional research is needed on gender nonconforming youth; in this meta-analysis, no studies reported data for gender nonbinary youth [15].

Follow-up services, such as outpatient psychiatric care, are considered a critical piece of transitioning away from inpatient care. However, studies of outpatient services’ impact on readmissions have had conflicting results. For example, in a study of commercially and publicly insured adults with schizophrenia and bipolar disorder, outpatient visits after discharge were associated with a lower rate of hospital readmission [28]. While other studies have also found outpatient visits were associated with lower readmission [29-31], several studies have found outpatient services associated with *higher* rates or risk of readmission [32-34], while one found no difference [35]. Given these conflicting findings, it is likely that outpatient care interacts with other factors to influence readmission. A study of case management services after discharge found that timing was so critical that each 1 day of delay indicated a 0.4% increased likelihood for earlier readmission [25].

While nearly all research has focused on adult populations, a recent study looked more closely at youth’s outpatient care, linking it to a lower likelihood of psychiatric readmission for youth overall. The relationship between outpatient care to readmissions differed by the length of hospital stay—youth with shorter hospital stays were more likely to be readmitted when they received aftercare. However, youth with longer stays and aftercare were less likely to be readmitted [34]. Another key factor was the intensity of aftercare services (eg, number of hours or sessions), but this has been understudied. Receiving more hours of aftercare, particularly day treatment, was associated with a lower risk of rehospitalization within 6 months [36].

### Intensive Outpatient Programs as a Solution to Escalating Need

Intensive outpatient (IOP) care, provided for multiple hours every week, is 1 option to reduce the number of patients at risk for readmissions for mental health conditions through the provision of appropriate step-down care for youth as they are discharged from the hospital [14,37]. A growing research base on youth’s IOP programs demonstrates significant symptom reduction and improved functioning [38-40].

Only 1 study to our knowledge has specifically assessed IOP services and readmission; in an analysis of all 11,473 adult IOP program services in Connecticut, individuals who completed at least a minimally adequate dosage of care had significantly lower rates of readmission [41]. Similarly, when an intensive short-term dynamic psychotherapy protocol was implemented for 50 adult patients with possible anxiety or somatization concerns, their ED visits reduced by 69% [42]. The preliminary research base on mental health IOP care effectiveness in reducing health care costs for adult clients is promising [43,44]. To our knowledge, no research has assessed IOP services and youth’s readmission. Additional research is needed, particularly on the role of intensive services in addressing the escalating youth’s mental health readmission rates.

### Present Evaluation

The purpose of this program evaluation was to assess (1) whether youth and young adult ED admission rates decreased during participation in a remote IOP program and (2) whether there are differences between youth and young adults in readmission rates. This evaluation is part of ongoing routine outcomes monitoring to identify opportunities for quality improvement in care. Identifying current readmission rates is necessary to determine need for future quality improvement, and investigating differences by age, gender, sexual orientation, race, and ethnicity will allow for tailoring resources as necessary.

## Methods

### Client and Program Characteristics

The data for this program evaluation come from *Charlie Health,* a national remote IOP program for adolescents and young adults with high acuity mental and behavioral health needs. Charlie Health was operational in 18 states during the data collection period for clients included in this analysis.

Charlie Health serves a high-acuity population of youth that commonly present with primary depression and anxiety, as well as numerous co-occurring mental and behavioral health challenges. Many clients present with significant histories of trauma and step down to IOP care from a higher level of care. Recognizing the challenges associated with committing to an IOP program that requires 9 hours of group participation and optional individual and family sessions, Charlie Health provides group options during daytime and evening hours. Clients are assigned to a group “track” that is reflective of identity (ie, lesbian, gay, bisexual, transgender, queer, intersex, or asexual; gender; or age) or primary presenting issue (ie, trauma or suicidal ideation). A group comprises three 50-minute group sessions for 3 days each week wherein clients are exposed to process, experiential (ie, art therapy), and skills groups. The latter is facilitated within an evidence-based practice shown to be effective with the primary issue (ie, dialectical behavioral skills for suicidal ideation). The ability to tailor groups to identity and issue is predicated on the importance of group cohesion to therapeutic change.

The current analyses included youth who were discharged from care between September 1, 2022, and November 30, 2022. In order to assess treatment efficacy in reducing ED admissions, inclusion criteria permit the use of client cases that completed treatment or experienced a treatment episode disruption (ie, disengagement and discharge against clinical advice). However, in order to ensure adequate treatment dosage, the Charlie Health evaluation team only included cases where clients completed at least 2 weeks of treatment and 6 IOP sessions. Optimal doses of therapy range between 4 and 26 hours [45]. Although scarce research has examined optimal doses for samples with severe mental disorders [45], clients require longer treatments for a larger magnitude of change [46]. The inclusion criteria for this study were based on an early systematic review suggesting 18 hours of therapy for the average patient to reach positive outcomes [47]. As such, the results of the following analyses can only be generalized to clients that completed intake and 3-month postdischarge surveys and met the minimal engagement threshold (N=735). This program evaluation focuses on quantitative survey questions regarding ED admissions; other qualitative client responses are provided in other publications [48].

### Ethics Approval

This research was approved by the Florida State University institutional review board as “non-human subjects research” given its primary purpose of conducting program evaluation (STUDY00003364).

### Data Collection Procedures

The data came from treatment surveys that were distributed to clients during their first remote IOP session (“intake”) and 3 months after their last IOP session (“3-month postdischarge”). Data were collected using an electronic survey that is distributed to clients by Charlie Health staff. When clients arrived at their first remote IOP session, they were sent to a room where a staff member provided them with a link to the survey. At 3 months, clients were emailed and texted a link to the survey and provided reminders to complete the survey for up to 4 weeks, at which point the survey closed.

### Measures

#### Overview

Clients were asked at intake and 3 months after discharge if they were admitted to an ED within the previous 30 days. Clients were then asked to select from a list of reasons why they were admitted.

#### General ED Admissions

To compare intake and 3-month postdischarge changes in ED admissions, the dichotomous question about whether clients have been admitted to an ED in the past 30 days was used to assess pre- or postchange.

#### Mental Health–Related ED Admissions

Mental health–related ED admissions were operationalized as clients that reported an ED admission and provided a reason related to mental or behavioral health. Reasons included suicidal thoughts, suicide attempts, physical altercation, self-harm, substance abuse, or eating disorder. Clients that provided no reason or selected the “other” option were classified as “general ED admissions.”

### Data Preparation

In order to conduct the analyses for this evaluation, a new variable was created to identify clients that had mental health–related admissions (defined below). In addition, demographic characteristics were used to assess significant differences on intake ED admissions that would need to be considered in the main analyses. In order to create this variable, 2 variables were combined: (1) a general question about ED admissions in the last 30 days (0=no and 1=yes) and (2) a reason for admission (7-item multiple-choice question where responses 1 through 6 listed mental or behavioral health reasons and 7 is an “other” option). First, the general ED admission question was transformed to remove cases with missing data on this question. Second, the ED admission “reason” variable was recoded into a dichotomous variable: 0=general ED admission and 1=mental health–related reason. The variables were combined wherein clients with a score of “0” were classified as “no ED admission,” clients with a score of “1” were classified as a “general ED admission,” and clients with a score of “2” were classified as having a “mental health–related ED admission.”

### Data Analysis Strategy

#### Descriptive Statistics

Descriptive statistics were run to better understand the distribution of demographic characteristics including age group (adolescent and young adult), gender identity, sexual orientation, race, and ethnicity. Descriptive information was also provided on reasons for ED admissions for those clients that reported an intake or 3-month postdischarge ED admission and includes disclosed information about the reason (intake: n=177 and 3 months after discharge: n=88).

#### Outcomes Analysis

McNemar tests were used to assess significant change in general and mental health–related ED admissions from intake to 3 months after discharge. McNemar test was designed to assess differences in proportions between 2 paired samples of data, such as pre- and posttest study designs where data are collected from the same subjects before and after an intervention [49].

## Results

### Sample Characteristics

During this study’s period, 1714 clients were discharged from programming. Of the total population of discharging clients, 71.4% (1223/1714) met the engagement threshold of attending at least 6 sessions in programming. Of clients who did not meet the engagement threshold, 71.3% (350/491) were discharged within a week of starting IOP programming.

### Missing Data

Nearly two-thirds (735/1223, 60.1%) of clients who reached the minimum threshold for engagement completed a discharge survey, resulting in a final sample size of 735 clients. For the analysis of recurrence of ED visits at 3 months after discharge, clients were only included in the 3 months sample if they (1) reported an ED visit in the 30 days prior to admission to programming, and (2) completed a 3-month postdischarge survey. Of the 177 clients who met the former criteria, 88 completed a 3-month postdischarge survey (response rate=49.7%). Due to the voluntary nature of the surveys for quality improvement, many client responses on demographics were missing. Missing data for demographic variables ranged from 3.9% (29/735) missingness on age to 38% (279/735) missingness on ethnicity. Cases missing data were deleted listwise.

A series of analyses compared those included in analyses with those who were discharged during the same period prior to completing 6 sessions or without completing a discharge survey. There were no significant differences between the 2 groups regarding gender (*χ* ^2^_783_=5.2, *P*=.07), sexual orientation (*χ*^2^_767_=1.9, *P*=.59), or ethnicity (*χ* ^2^_534_=1.0, *P*=.62). Significant differences were detected regarding race (*χ* ^2^_767_=15.2, *P*=.004) and age (mean 1.11, 95% CI –1.62 to –0.56 years; 2-tailed *t*_1261.63_=–4.03, *P*<.001). However, the effect size was small for both (race: ϕ=0.16 and age: Cohen *d*=0.22).

### Client Demographics

The median age of client cases in this sample was 18.25 (SD 4.94) years, wherein 57.6% (407/707) of the sample were adolescents (aged 11-17 years) and 42.4% (300/707) were young adults (older than the age of 18 years). Almost half (329/687, 47.9%) the sample identified as female, followed by male (196/687, 28.5%), and nonbinary (65/669, 9.7%). More than one-third (248/635, 39.1%) of the sample identified as “heterosexual or straight,” nearly a quarter (137/635, 21.6%) identified as “bisexual,” 10.9% (80/635) identified as “pansexual,” and 26.8% (170/635) identified as some other sexual orientation identity (eg, gay, lesbian, or queer). The majority (380/481, 79%) of the sample identified as White, followed by Black (48/481, 10%), Indigenous People Around the World (9/481, 1.9%), and Asian (9/481, 1.9%), while 7.2% (35/481) identified as some other racial identity. Nearly a quarter (112/455, 24.6%) of the sample identified as Hispanic, Latino, or Spanish Origin, while 69.2% (315/455) did not and 6.2% (28/455) identified as some other ethnic identity. Table 1 shows the distribution of mental and behavioral health reasons for ED admissions at intake and 3 months after discharge.

**Table 1:** Reasons for ED^a^ admissions at intake and 3 months after discharge.

Variable			Intake		3 months after discharge
Reason provided, n/N (%)			177/696 (25.4)		11/88 (12.5)
**Reason for ED admission (intake: n=177; 3 months after discharge: n=11), n (%)**					
	Suicidal thoughts	58 (34.3)		3 (27)	
	Suicide attempt	51 (30.2)		1 (9)	
	Physical altercation	6 (3.6)		0 (0)	
	Self-harm	17 (10.1)		0 (0)	
	Substance use	5 (3)		0 (0)	
	Eating disorder	7 (4.1)		2 (18)	
	Other	25 (14.8)		5 (45)	
	*Mental health related*	144 (81.4)		6 (54.5)	

^a^ED: emergency department.

### Demographic Correlates of Readmission

There were no statistically significant differences found on pretreatment ED admissions by age group, with a similar proportion of adolescent clients (111/401, 27.2%) reporting pretreatment ED admissions compared to young adults (66/295, 22.4%; *χ* ^2^_1_=2.5, *P=*.11). To investigate potential differences in change in ED visits over time by developmental stage, an improvement variable was created by subtracting the 3 months follow-up admission scores (0=no and 1=yes) from the pre-ED admission score (0=no and 1=yes). Clients with a score of “0” were classified as “not improved,” while clients with a score of “1” were classified as “improved,” representing cases that reported a preadmission ED visit and no ED admission at 3 months after discharge. The sample was restricted to only those clients that reported an intake ED admission and had completed a 3-month postdischarge survey (n=88). The results of the chi-square analysis indicated that there were no significant differences in the proportion of adolescent clients (41/50, 82%) and young adult clients (36/38, 95%) that improved from intake to 3 months after discharge (*χ* ^2^_1_=3.2, *P=*.07). Furthermore, there were no other significant differences found by demographic characteristics of ethnicity, race, gender, or sexual orientation (all *P*>.05; Table 2).

**Table 2:** Other demographic differences in ED^a^ admissions at intake and improvement.

Variable			ED admissions at intake											Improvement in admissions between intake and 3 months follow-up								
			No, n/N (%)		Yes, n/N (%)	*χ*^2^ (*df*)			*P* value			No, n/N (%)				Yes, n/N (%)		*χ*^2^ (*df*)			*P* value	
**Ethnicity**							0.4 (1)			.53									3.2 (1)			.07
	Hispanic or Latino	23/32 (73)		9/32 (27)							4/40 (10)				36/40 (90)							
	Not Hispanic or Latino	79/104 (76)		25/104 (24)							3/9 (33)				6/9 (67)							
**Race**							0.3 (1)			.58									1.1 (1)			.29
	Black, Indigenous, Asian, or other	73/94 (78)		21/94 (22)							2/8 (25)				6/8 (75)							
	White	266/355 (74.9)		89/355 (25.1)							5/45 (11)				40/45 (89)							
**Gender**							1.8 (2)			.40									0.5 (2)			.79
	Woman	236/310 (76.1)		74/310 (23.9)							4/40 (10)				36/40 (90)							
	Man	138/182 (75.8)		44/182 (24.2)							3/19 (16)				16/19 (84)							
	Nonbinary	99/141 (70.2)		42/141 (29.8)							2/20 (10)				18/20 (90)							
**Sexual orientation**							0.2 (3)			.98									2.4 (3)			.49
	Heterosexual	168/230 (73)		62/230 (27)							1/25 (4)				24/25 (96)							
	Bisexual	98/132 (74.2)		34/132 (25.8)							3/21 (14)				18/21 (86)							
	Pansexual	57/77 (74)		20/77 (26)							1/8 (13)				7/8 (88)							
	Other sexual orientation	120/160 (75)		40/160 (25)							4/22 (18)				18/22 (82)							

^a^ED: emergency department.

### General ED Admissions

McNemar test was used to compare ED admissions data from the same clients at 2 time points, including intake and at 3 months after discharge. The findings of the McNemar test indicated ED admissions significantly decreased from intake to 3 months after discharge, such that 87% (77/88) of clients with a history of ED admissions reported no ED admissions in the 3 months after discharge from treatment (*P<*.001; Table 3).

**Table 3:** Change in ED^a^ admissions from intake to 3 months after discharge across the whole sample (χ 21=52.2, *P*<.001).

Improved between intake or 3 months after discharge				3-month postdischarge ED admission				Total
				No		Yes		
**Pre-ED admission**								
	**No**							
		Count (n=223), n (%)	214 (96)		9 (4)		223 (100)	
		Standard residual	0.4		–0.4		N/A^b^	
	**Yes**							
		Count (n=88), n (%)	77 (87)		11 (12)		88 (100)	
		Standard residual	–0.6		0.6		N/A	
Total (n=311), n (%)				291 (93.6)		20 (6)		311 (100)

^a^ED: emergency department.

^b^N/A: not applicable.

### Mental Health–Related ED Admissions

Findings from the McNemar test indicate that mental health–related ED admissions significantly decreased from intake to 3 months after discharge such that 94% (65/69) of clients with a history of mental health–related ED admissions reported no mental health–related ED admissions in the 3 months after discharge from treatment (*P<*.001; Table 4).

**Table 4:** Change in mental health–related ED^a^ admissions from intake to 3 months after discharge (χ 21=38.8, *P*<.001).

Improved between intake or 3 months after discharge				3-month postdischarge MH^b^-related ED admission				Total
				No	Yes			
**Pre–MH-related ED admission**								
	**No**							
		Count (n=236), n (%)	226 (95.8)		10 (4)	236 (100)		
		Standard residual	0.1		–0.3	N/A^c^		
	**Yes**							
		Count (n=69), n (%)	65 (95)		4 (6)	69 (100)		
		Standard residual	–0.1		0.5	N/A		
Total (n=305), n (%)				291 (95.4)	14 (5)		305 (100)	

^a^ED: emergency department.

^b^MH: mental health.

^c^N/A: not applicable.

## Discussion

### Principal Findings

Youth and young adults reported a significant decrease in mental health ED admissions between intake and 3 months after discharge from Charlie Health IOP programming, with only 6% (4/69) of clients with a recent mental health ED admission at intake reporting an ED admission at 90-day follow-up. This sample includes gender and sexual minority youth missing from previous research [15] and represents a sample with co-occurring disorders and acute needs that come with high risk of readmission [23,25]. Recent research found that less than half of youth and young adults who visit the ED for mental health reasons receive follow-up care, with the authors issuing a call for improved engagement with outpatient mental health providers to increase follow-up rates [50]. This study documented positive outcomes among youth and young adults who did engage in IOP care following ED visits. This program evaluation is the first to our knowledge to examine readmission to ED following mental health IOP among youth specifically. In this investigation, youth and young adults were equally likely to have an ED admission at intake into the IOP program, and both age groups made similar improvements. Furthermore, there were no differences in the likelihood of reporting an ED admission by gender, sexual orientation, race, or ethnicity, and the reduction in ED admissions at 3 months after discharge was similar across all demographic subgroups.

### Limitations

This study represents a formative evaluation study and has significant limitations to be addressed in future research. Most notably, it is not possible to include a matched control group in this program evaluation, as Charlie Health works to provide care to all youth and young adults with intensive needs as quickly as possible. We cannot conclusively determine that IOP care causes the drop in ED admissions without comparing an IOP sample to a matched sample that does not participate. This evaluation relies on self-report data and excludes youth and young adults who did not complete the 3-month postdischarge assessment, which may include other youth or young adults who required a readmission. Furthermore, this study is limited to youth and young adults who enrolled in care. As many clients with mental health concerns do not access follow-up care following discharge from an inpatient admission [51], these youth may have protective factors that enabled them to enroll in care and prevented readmission.

### Comparison With Prior Work

The Charlie Health 3-month mental health ED readmission rate of 6% (4/69) is substantially lower than those reported in broader community samples, such as the 13.2% psychiatric pediatric readmission rate found in the meta-analysis by Edgcomb et al [15] or the 15.6% psychiatric readmission rate for young adults nationally [16]. It is also higher than the 30-day rate in broader studies of outpatient care, such as the 22.2% psychiatric readmission rate among young adults in outpatient treatment for being bipolar [27].

Further, 1 explanation for this relatively low readmission rate is that this sample is restricted to those who received an adequate dose of care, and studies that include participants who drop out are likely to have higher readmission rates. Another explanation is that a more intensive or higher dose of services meet acute needs [46] following discharge, and the level of care is appropriate for reducing readmissions for youth as it is for adults [41]. The telehealth format may also contribute. It may be that telehealth has lower barriers to engagement than in-person services, such as transportation [52,53], and so youth and young adults are able to participate even when they face escalating symptoms or functioning challenges that could otherwise lead to crises requiring inpatient care.

If the 6% (4/69) Charlie Health readmission rate reflected a reduction from the average 13.2% (10,076/83,361) youth readmission rate [15], this would represent a 56.1% reduction in psychiatric readmissions and associated cost savings. This is similar to the 58% cost ratio found for youth with physical health concerns participating in an IOP program, compared to youth in the control group, with total costs reduced by US $10,258 per child-year in 2011-2013 [54].

### Conclusions

A quarter (177/696, 25.4%) of Charlie health clients in this sample began IOP services shortly following an ED admission, representing a step down in care meant to address continued acute needs. Evaluating effectiveness at reducing future ED admissions is necessary for Charlie Health to identify if there is a need to offer more or less intensive services. These results suggest that clients experience a significant reduction in ED admissions between intake and 3 months after IOP treatment. The similar outcomes between age groups indicate that the current tracks of programming are equally meeting the needs of both youth and young adults, as well as the needs of youth from a variety of different demographic backgrounds.

This promising preliminary finding demonstrates the need for a full return-on-investment analysis for intensive mental health services for youth and young adults. Potential cost-savings to health care payers of intensive mental health services include not only reduced hospital readmission but reduced health care expenses for family members [55], reduced general health care costs [56], and reduced risk of suicide attempts with the associated cost of care at the time of attempt [57] and increased cost of care during the following year [19]. The potential benefits to society of services that meet the acute mental health needs of youth and young adults are far greater, including improved quality of life [44] and earnings [58], as well as reduced absenteeism [59]. Providing access to the appropriate level of mental health care for youth and young adults in need is a crucial first step in reducing the overuse of EDs for mental health crises.

## Abbreviations

ED: emergency department
IOP: intensive outpatient

## Data Availability

Owing to the quality improvement nature of this study, the data sets analyzed contain information that could compromise participants’ privacy and participants did not agree to their data being shared publicly, so supporting data are not available.
